# Temporal Association of Reduced Serum Vitamin D with COVID-19 Infection: Two Single-Institution Case–Control Studies

**DOI:** 10.3390/nu14132757

**Published:** 2022-07-02

**Authors:** Diviya Gupta, Sahit Menon, Michael H. Criqui, Bryan K. Sun

**Affiliations:** School of Medicine, University of California San Diego, La Jolla, CA 92093, USA; digupta@health.ucsd.edu (D.G.); s1menon@health.ucsd.edu (S.M.); mcriqui@health.ucsd.edu (M.H.C.)

**Keywords:** COVID-19, vitamin D, 25(OH)D, case–control study

## Abstract

(1) Background: Vitamin D supplementation has been proposed for the prevention and treatment of COVID-19, but it is not clear if reduced serum vitamin D predisposes individuals to COVID-19 and/or is a secondary consequence of infection. This study assessed the temporal association between serum vitamin D and COVID-19 with two single-institution case–control studies through the University of California San Diego (UCSD) Health System. (2) Methods: This study included patients who tested positive for COVID-19 from 1 January to 30 September 2020 with serum 25-hydroxy-vitamin D (25(OH)D) measured within 180 days of diagnosis. Patients were separated based on whether 25(OH)D was measured before (*n* = 107 cases, 214 controls) or after (*n* = 203 cases, 406 controls) COVID-19 diagnosis. COVID-19 infection status was the outcome variable in the pre-diagnosis study, whereas serum 25(OH)D level was the outcome variable in the post-diagnosis study. (3) Results: Serum 25(OH)D levels were not associated with the odds of subsequent COVID-19 infection (OR 1.0, 95% CI: 1.0 to 1.0, *p* = 0.98). However, COVID-19-positive individuals had serum 25(OH)D measurements that were 2.7 ng/mL lower than the controls (95% CI: −5.2 to −0.2, *p* = 0.03). (4) Conclusions: In our study population, serum 25(OH)D levels were not associated with the risk of acquiring COVID-19 infection but were reduced in subjects after COVID-19 infection. These results support the possibility that reduced serum 25(OH)D is a consequence and not a cause of COVID-19 infection.

## 1. Introduction

SARS-CoV-2, the coronavirus that causes COVID-19, has claimed over 6 million lives globally. In addition to vaccination efforts, there has been widespread public interest in complementary measures to mitigate the risk of viral infection and mortality. Vitamin D, available as an inexpensive supplement, gained extensive attention for its potential role in the prevention and treatment of COVID-19. However, the relationship between vitamin D and COVID-19 is unclear. 

Mechanistically, vitamin D may enhance the immune response to SARS-CoV-2 in several ways. Vitamin D boosts innate immunity by augmenting the expression of human cathelicidin antimicrobial peptide (CAMP) [1] in lung and skin epithelial cells [2], which has been shown to attenuate the infectivity and viability of viruses [3]. In addition, vitamin D prevents excessive adaptive immune responses [4]. Because systemic inflammatory responses have been associated with respiratory distress and mortality in patients with severe COVID-19 [5], it has been proposed that vitamin D supplementation could mitigate these harmful inflammatory reactions. 

Some medical experts and public figures have recommended vitamin D supplementation for COVID-19 [6], using serum 25-hydroxy-vitamin D (25(OH)D), a standard laboratory serum marker of vitamin D stores, as a primary biomarker for vitamin sufficiency. Recommendations for supplementation have particularly been endorsed for populations with the most elevated COVID-19 risk and who also have higher rates of vitamin D deficiency, including the elderly, individuals with chronic diseases, darker-complected individuals such as African Americans, and those who live at high latitudes [7].

The studies that have evaluated the correlation between serum vitamin D and COVID-19 have shown mixed results. Some reports have concluded that there is an association between vitamin D deficiency and increased susceptibility to COVID-19 infection [8,9], but others have not [10,11]. Other studies, focusing on different endpoints, have found that 25(OH)D deficiency is correlated with a higher risk of intensive care unit admission, ventilation dependency, and a lower survival rate [12,13,14,15,16]. Beneficial outcomes from vitamin D supplementation trials have been reported [17], although these therapeutic studies have not always been conducted in randomized groups [18] and have had modest sample sizes. It has been postulated that any beneficial effect of vitamin D on severe COVID-19 could be masked by the effect of other adjunctive treatments such as dexamethasone [19]. Highly powered, randomized controlled trials will be needed to definitively test for causality.

The differences in conclusions among the published results may be caused, at least in part, by significant methodological differences. Some reports examined serum 25(OH)D based on country-wide averages [20,21] or inferred vitamin D status based on geographic latitude [22] and did not directly assess serum vitamin D in individual subjects. Other studies used vitamin D results that were measured years prior to COVID-19 testing [23], which may have resulted in inaccurate representations because 25(OH)D levels can change significantly with time [24] and season [25]. Finally, some reports assessed 25(OH)D levels drawn prior to COVID-19 diagnosis [8], while others relied on measurements taken after diagnosis [12,26,27], and others included both [28]. Because severe illness itself can cause the rapid reduction of serum 25(OH)D [29], the timing of laboratory measurement is critical: vitamin D deficiency may predispose individuals to COVID-19, but it is also possible that COVID-19 infection can reduce 25(OH)D.

In this report, we sought to address these methodological differences by examining the temporal correlation between serum 25(OH)D and COVID-19 infection. We performed two complementary single-center studies examining patients testing positive for COVID-19 between 1 January 2020 to 30 September 2020 in the University of California San Diego (UCSD) Health system who had a serum 25(OH)D assessment within 180 days of diagnosis. These dates capture most COVID-19 cases in the UCSD Health system from the onset of the COVID-19 pandemic until the initiation of vaccinations.

In the first study, we used a case–control design to compare the 25(OH)D levels drawn prior to COVID-19-positive diagnosis with COVID-19-negative controls matched by age, sex, body mass index (BMI), diabetes, hypertension, time from vitamin D draw, and the season that the 25(OH)D test was performed. In the second study, we applied a case–control design to assess serum 25(OH)D levels drawn after COVID-19 diagnosis, applying the same matching criteria. Finally, we performed a subgroup analysis of the second study to specifically examine COVID-19 patients whose disease was severe enough to require hospitalization, comparing them against a matched hospitalized cohort that was COVID-19-negative.

Our overarching approach was based on the reasoning that if vitamin D deficiency increased susceptibility to COVID-19 infection, then serum 25(OH)D levels drawn prior to diagnosis would be significantly lower in cases vs. controls. However, if 25(OH)D was lower in COVID-19 cases only if measured after diagnosis, then COVID-19 infection itself may have led to a reduction in vitamin D levels. The null hypotheses were that there was no correlation between 25(OH)D levels and COVID-19 in either study. Finally, by focusing on the subgroup of patients requiring hospitalization for COVID-19 and comparing it to a matched hospitalized cohort, we sought to examine if any correlation between 25(OH)D and COVID-19 was amplified among patients affected by severe forms of COVID-19 illness.

## 2. Materials and Methods

The UCSD Institutional Review Board approved this study and approved a waiver for informed consent based on the requirements outlined in the Code of Federal Regulations on the Protection of Human Subjects (45 CFR 46). All data collection and analysis were performed in accordance with relevant guidelines and regulations. Data were collected for all subjects who tested for COVID-19 through the UCSD Health system from 1 January 2020 to 30 September 2020 (*n* = 6050), capturing an interval from the onset of local infections to the time prior to the initiation of COVID-19 vaccinations.

**Figure 1:**
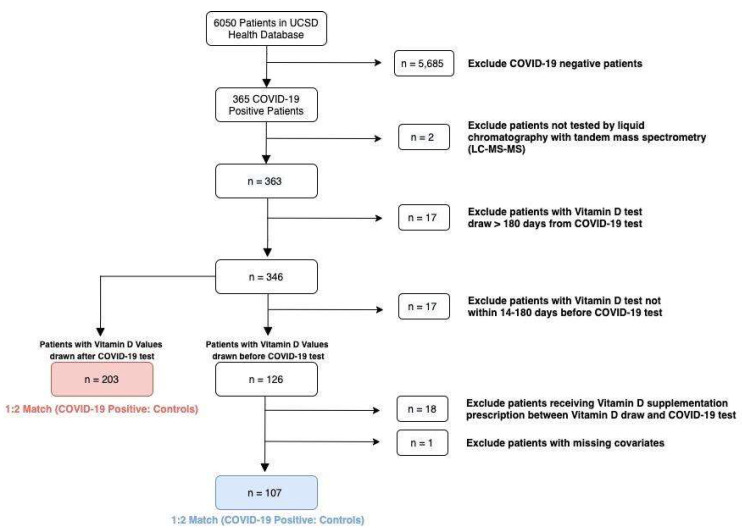
Flow diagram showing the selection of study subjects and controls.

For primary analysis (Figure 1), cases were identified as patients who had serum 25(OH)D drawn within 180 days of COVID-19 diagnosis (*n* = 346). Seventeen patients had serum 25(OH)D drawn within 14 days of COVID-19 diagnosis and were excluded to account for the SARS-CoV-2 incubation period and to minimize confounding by possible early manifestations of COVID-19 [30]. Additionally, 18 patients in the pre-diagnosis study received vitamin D supplementation after serum 25(OH)D testing, but before COVID-19 diagnosis; these subjects were excluded due to probable changes in serum 25(OH)D levels from supplementation. One patient was removed from the pre-diagnosis study due to incomplete data on matching covariates.

After exclusions, there were 107 cases in the pre-diagnosis study and 203 cases in the post-diagnosis study. Cases in both studies were matched 1:2 to COVID-19-negative controls by age, sex, BMI, diagnosis of diabetes (ICD-10 codes E11.0–E11.9), diagnosis of hypertension (ICD-10 code I10), the number of days between vitamin D draw and COVID-19 diagnosis, and the meteorological season of 25(OH)D laboratory draw. Patient ethnicity and skin phototype data were not available. Matching was performed using nearest-neighbor matching based on Mahalanobis distance.

For the pre-diagnosis study, a case–control analysis was performed with 25(OH)D as the independent variable and COVID-19 infection status as the dependent variable. A conditional logistic regression model was performed to estimate the odds ratio (OR) and 95% confidence interval (95% CI) for serum 25(OH)D as a continuous variable. Results are reported as the change in odds of COVID-19 positivity for every 1 ng/mL increase in 25(OH)D.

For the post-diagnosis study, a case–control analysis was performed in which COVID-19 infection status served as the binary independent variable to assess serum 25(OH)D levels as the outcome. An ordinary least squares (OLS) regression model was performed to estimate the change in serum 25(OH)D per ng/mL associated with COVID-19 positivity.

In a subset analysis of the second study, serum 25(OH)D levels in hospitalized COVID-19 patients were compared to COVID-19-negative hospitalized patients. Here, cases were defined as patients who had serum 25(OH)D drawn up to 180 days after inpatient admission due to COVID-19 (*n* = 120). Matching criteria were identical to other analyses except for two changes. First, subjects were matched by the number of days between vitamin D draw and COVID-19 hospitalization, not COVID-19 diagnosis, and second, length of hospital stay was included as an additional matching covariate to promote the comparison of hospitalized patients with similar disease severities [31]. An OLS regression model was performed to estimate the change in serum 25(OH)D per ng/mL associated with COVID-19 illness.

Analyses were performed using RStudio software, version 1.3.959. Continuous covariates are reported as mean ± standard deviation (SD) and compared using unpaired *t*-tests; categorical variables are reported as numbers and percentages and compared using Chi-square tests. The significance level for all analyses was set to a two-sided *p*-value < 0.05.

Subjects or the public were not involved in the design, conduct, reporting, or dissemination plans of this study.

## 3. Results

### 3.1. Serum 25(OH)D Was Lower in COVID-19 Subjects Tested after, but Not before Diagnosis

Baseline characteristics of the study cohorts are shown in Table 1 and Table 2. Covariates consisted of established risk factors for COVID-19 susceptibility [32] that included age, sex, obesity, and medical comorbidities. In addition, due to seasonal variation in serum vitamin D levels, control subjects were matched by the meteorological season that the serum vitamin D was measured, as well as length of the delay between serum vitamin D assessment and COVID-19 testing. Matched study populations were balanced between groups.

For subjects in which 25(OH)D serum levels were assessed prior to COVID-19 testing, the mean serum 25(OH)D was 35.5 ng/mL (SD 13.7) for cases and 35.4 ng/mL (SD 13.8) for controls. A one-unit increase in serum 25(OH)D did not affect the odds of contracting COVID-19 (OR 1.0, 95% CI 1.0 to 1.0, *p* = 0.98). These data revealed no significant association between serum 25(OH)D and the odds of subsequent COVID-19 positivity.

In contrast, in subjects for whom 25(OH)D was measured after diagnosis, subsequent assessment of 25(OH)D showed a mean serum 25(OH)D of 30.5 ng/mL (SD 15.5) for cases and 33.2 ng/mL (SD 15.7) for controls. COVID-19 positivity was associated with serum 25(OH)D levels that were lower by 2.7 ng/mL on average (95% CI −5.2 to −0.2, *p* = 0.03) (Table 3 and Table 4). These data indicated that COVID-19-positive subjects showed a significant reduction in 25(OH)D compared to matched COVID-19-negative subjects.

Continuous covariates are reported as mean ± standard deviation (SD) and compared using unpaired *t*-tests. Categorical variables are reported as numbers (no.) and percentages (%) and compared using Chi-square tests. A *p*-value < 0.05 was considered a significant difference for covariates. Season dates were defined by the meteorological seasons within the study interval.

Serum 25(OH)D for cases and controls are reported as mean levels in ng/mL ± standard deviation (SD). In the pre-diagnosis study, a conditional logistic regression was performed in which the independent variable is continuous (i.e., serum 25(OH)D level) and the dependent variable is binary (i.e., COVID-19 infection status).

In the post-diagnosis study, an ordinary least squares regression was performed, in which the independent variable is binary (i.e., COVID-19 infection status) and the dependent variable is continuous (i.e., serum 25(OH)D level). Abbreviations: CI = confidence interval; 25(OH)D = 25-hydroxy vitamin D.

### 3.2. Reduced 25(OH)D in COVID-19-Positive Hospitalized Patients Compared to a COVID-19-Negative Hospitalized Cohort

It has been reported that vitamin D deficiency may correlate to severe outcomes from COVID-19 infection. To assess for potential correlation between serum 25(OH)D and severe COVID-19 infection, a subgroup analysis was performed to compare COVID-19 subjects requiring hospitalization against a COVID-negative hospitalized control group. To promote matching patients with comparable disease severity, control patients were also matched by the length of hospital stay, an indicator of disease severity [31]. Baseline characteristics of the matched analysis for the hospitalized cohort are shown in Table 5. No significant differences were observed for matching characteristics between cases and controls.

Continuous covariates are reported as mean ± standard deviation (SD) and compared using unpaired *t*-tests. Categorical variables are reported as numbers (no.) and percentages (%) and compared using Chi-square tests. A *p*-value less than 0.05 was considered a significant difference for covariates. Season dates were defined by the meteorological seasons within the study interval.

Within a 180-day window following inpatient admission, Table 6 shows that COVID-19-positive hospitalized cases had a mean 25(OH)D of 23.9 ng/mL (SD 13.5), while COVID-19-negative hospitalized controls had a mean 25(OH)D level of 27.3 ng/mL (SD 15.4). Thus, patient hospitalization due to COVID-19 infection was associated with 3.3 ng/mL (95% CI −6.3 to −0.4, *p* = 0.03) lower serum 25(OH)D levels compared to hospitalized COVID-19-negative patients (Table 6). These data indicated that COVID-19-positive hospitalized subjects showed a significant reduction in 25(OH)D compared to COVID-19-negative hospitalized subjects.

Serum 25(OH)D for cases and controls is reported as mean levels in ng/mL ± standard deviation (SD). An ordinary least squares regression was performed in which the independent variable is binary (i.e., hospitalization due to COVID-19 or hospitalization due to another cause) and the dependent variable is continuous (i.e., serum 25(OH)D level). Abbreviations: CI = confidence interval; 25(OH)D = 25-hydroxy vitamin D.

## 4. Discussion

Defining the temporal association between serum vitamin D and COVID-19 infection provides a basis for evaluating the potential use of vitamin D supplementation to prevent COVID-19 infection and/or mitigate disease severity. Our single-center study found that COVID-19 infection and hospitalization were associated with lower serum vitamin D levels drawn after diagnosis or hospital admission. However, serum 25(OH)D levels did not affect the odds of initially testing positive for COVID-19, indicating that lower vitamin D was not a risk factor for COVID-19 infection in our study population. Among the published studies, some reports have proposed that vitamin D insufficiency, defined as serum concentrations 20–30 ng/mL, or vitamin D deficiency, defined as concentrations <20 ng/mL, may be risk factors for COVID-19 infection [33]. To explore this possibility, we also stratified our pre-diagnosis subjects into these categories. We found no statistically significant association with increased odds of COVID-19 with either vitamin D insufficiency or deficiency compared to the sufficient (>30 ng/mL) reference group. Viewed together, our results are consistent with other reports that identified lower 25(OH)D in association with COVID-19 [8,12,16,34], but suggest that lower 25(OH)D levels may be an outcome of COVID-19 infection rather than a cause of it.

The relationship between vitamin D levels and medical conditions is complex. In certain contexts, including a study that analyzed the relationship between 25(OH)D concentration and non-severe community-acquired pneumonia, there was no difference in serum (OH)D levels during the acute phase and up to 90 days after recovery [35]. Similarly, a study of 14 patients in the acute phase response to malaria demonstrated that vitamin D levels remained unaffected over the course of hospital stay and 2–6 weeks after discharge [36]. In contrast, in studies on pancreatitis, vitamin D has been shown to have statistically significant decreases due to inflammatory processes [37,38]. A systematic review cautioned against the notion that inflammatory conditions generate rapid decreases in vitamin D, partly because its findings illustrated the possibility of heterogeneity; however, many studies still showed a reduction in vitamin D following inflammatory insult [39]. Given the plausible relationship between inflammatory conditions and changes in vitamin D levels, reverse causality may explain some of the changes in vitamin D levels, and that preexisting vitamin D status alone is not solely responsible [40].

Our findings are consistent with prior reports that examined the relationship between vitamin D and respiratory illnesses. While vitamin D deficiency has been associated with an increased risk of respiratory infections [41], acute illness itself can also reduce serum 25(OH)D through fluid shifts, the depletion of serum binding proteins, and renal wasting [29]. Acute inflammation following surgery has been associated with a reduction in serum 25(OH)D within 48 h [42]. Inflammatory mediators lead to an increase in the activity of CYP24A1 and CYP27B1, enzymes that metabolize vitamin D pathway compounds [43]. Vitamin D metabolism is dysregulated in patients with asthma and chronic obstructive pulmonary diseases, leading some investigators to suggest that the relationship between airway inflammation and vitamin D deficiency is bidirectional [44]. These findings support the biological plausibility that elevated rates of vitamin D deficiency observed in subjects infected by COVID-19 could be, at least in part, secondary to the respiratory infection itself [40].

Our study has several strengths. First, our data from a single institution directly assessed 25(OH)D and COVID-19 laboratory results using uniform, standardized assays, minimizing the significant variations that can occur between different testing methodologies [45]. Second, our restriction of 25(OH)D values to a 180-day window, matching controls by season, and matching by the time interval between 25(OH)D and COVID-19 testing addresses important time-dependent effects that affect 25(OH)D levels [23,25]. Third, our stratification of 25(OH)D data from the same institutional population into subjects who were tested before vs. after COVID-19 diagnosis allowed us to assess the temporal relationship between vitamin D and COVID-19.

Our study also has several limitations. The retrospective design does not allow for the determination of causality, and our sample size is not powered to detect smaller, but potentially significant, correlations. Additionally, although we matched baseline characteristics between cases and controls, other unaccounted confounders could have affected the results. Data on the racial identity of our study subjects was incomplete, which did not allow us to include this factor in cohort matching. Racial disparities in COVID-19 illness have been observed, with one retrospective study finding that a positive COVID-19 test was associated with lower vitamin D levels in Black but not White individuals [8]. Our health system’s catchment area (San Diego County) has a lower Black population (~5.5%) than the U.S. national average (~13.4%) and has a relatively high level of sun and UV exposure. UV exposure may provide a protective effect against COVID-19 both through vitamin D-dependent and -independent mechanisms [46]. Therefore, the results from our analysis may differ from other study populations and do not argue against the potential utility of vitamin D supplementation for specific populations.

Ultimately, intervention trials could provide the most conclusive insight to the therapeutic value of vitamin D supplementation both prior to and after COVID-19 infection. Early reports have shown mixed results: A small (*n* = 76) randomized trial indicated that oral calcifediol supplementation reduced the need for intensive care unit admission in COVID-19-infected subjects [17], though the trial was not placebo-controlled and did not measure baseline or post-treatment serum vitamin D levels. By contrast, a randomized, double-blind, placebo-controlled trial on hospitalized COVID-19 patients found no benefit to a single 200,000 IU dose of vitamin D3 on the length of hospital stay [47]. Viewed together with the results from our study and taken in context with other published studies to date, we recommend caution in the therapeutic expectations for vitamin D supplementation in the prevention of COVID-19.

## 5. Conclusions

We performed two complementary case–control studies at UCSD Health to evaluate the temporal association of serum vitamin D and COVID-19 infection, examining 25(OH)D levels drawn before or after COVID-19 diagnosis. Our main finding is that serum 25(OH)D drawn before COVID-19 diagnosis did not differ between cases and controls, but serum 25(OH)D drawn in subjects who tested positive for COVID-19 was significantly lower than matched controls. This result was even more pronounced in subjects with severe COVID-19 infection requiring hospitalization. These results support the possibility that lower serum vitamin D levels may not predispose individuals to COVID-19 infection, but may be a consequence of it.

## Figures and Tables

**Table 1:** Characteristics of the COVID-19 study population and controls: serum 25(OH)D measured before COVID-19 diagnosis.

Characteristic	COVID-19-Positive (*n* = 107)	COVID-19-Negative (*n* = 214)	*p*-Value
**Age (years) ± SD**	51.2 ± 16.0	52.6 ± 15.3	0.49
**Sex—no. (%)**			
Male	53 (49.5)	106 (49.5)	1.00
Female	54 (50.5)	108 (50.5)	
**Body Mass Index ± SD**	27.8 ± 5.8	27.5 ± 5.4	0.67
**Diabetes—no. (%)**			
Yes	22 (20.6)	44 (20.6)	1.00
No	85 (79.4)	170 (79.4)	
**Hypertension—no. (%)**			
Yes	50 (46.7)	100 (46.7)	1.00
No	57 (53.3)	114 (53.3)	
**Days between vitamin D and**			
**COVID-19 test ± SD**	77.4 ± 39.9	77.7 ± 39.6	0.95
**Season—no. (%)**			
Winter (1 January–29 February)	36 (33.6)	72 (33.6)	
Spring (1 March–31 May)	33 (30.8)	68 (31.8)	0.98
Summer (1 June–31 August)	38 (35.5)	74 (34.6)	
Fall (1 September–30 September)	-	-	

**Table 2:** Characteristics of the COVID-19 study population and controls: serum 25(OH)D measured after COVID-19 diagnosis.

Characteristic	COVID-19-Positive (*n* = 203)	COVID-19-Negative (*n* = 406)	*p*-Value
**Age (years) ± SD**	52.7 ± 15.7	53.4 ± 15.2	0.60
**Sex—no. (%)**			
Male	123 (60.6)	246 (60.6)	1.00
Female	80 (39.4)	160 (39.4)	
**Body Mass Index ± SD**	28.0 ± 6.5	27.6 ± 5.9	0.44
**Diabetes—no. (%)**			
Yes	70 (34.5)	140 (34.5)	1.00
No	133 (65.5)	266 (65.5)	
**Hypertension—no. (%)**			
Yes	109 (53.7)	218 (53.7)	1.00
No	94 (46.3)	188 (46.3)	
Days between vitamin D and			
COVID-19 test ± SD	32.3 ± 40.4	32.3 ± 38.9	0.99
Season—no. (%)			
Winter (1 January–29 February)	-	-	
Spring (1 March–31 May)	42 (20.7)	82 (20.2)	
Summer (1 June–31 August)	124 (61.1)	251 (61.8)	0.98
Fall (1 September–30 September)	37 (18.2)	73 (18.0)	

**Table 3:** Pre-diagnosis—conditional logistic regression.

Predictor	Cases (*n* = 107) Serum 25(OH)D (ng/mL) ± SD	Controls (*n* = 214) Serum 25(OH)D (ng/mL) ± SD	Odds Ratio	95% CI	*p*-Value
Vitamin D (ng/mL)	35.5 ± 13.7	35.4 ± 13.8	1.0	1.0 to 1.0	0.98

**Table 4:** Post-diagnosis—ordinary least squares regression.

Predictor	Cases (*n* = 203) Serum 25(OH)D (ng/mL) ± SD	Controls (*n* = 406) Serum 25(OH)D (ng/mL) ± SD	Beta Estimate	95% CI	*p*-Value
COVID-19 Infection	30.5 ± 15.5	33.2 ± 15.7	−2.7	−5.2 to −0.2	0.03

**Table 5:** Characteristics of hospitalized COVID-19 study population and controls.

Characteristic	COVID-19-Positive (*n* = 120)	COVID-19-Negative (*n* = 240)	*p*-Value
**Age (years) ± SD**	55.9 ± 14.6	57.9 ± 14.0	0.22
**Sex—no. (%)**			
Male	82 (68.3)	165 (68.8)	0.94
Female	38 (31.7)	75 (31.3)	
**Body Mass Index ± SD**	28.1 ± 6.8	26.8 ± 6.3	0.07
**Diabetes—no. (%)**			
Yes	59 (49.2)	115 (47.9)	0.82
No	61 (50.8)	125 (52.1)	
**Hypertension—no. (%)**			
Yes	83 (69.2)	165 (68.8)	0.94
No	37 (30.8)	75 (31.4)	
**Days between vitamin D test and**			
**COVID-19 hospitalization ± SD**	21.0 ± 34.4	19.9 ± 30.0	0.76
**Season—no. (%)** Winter (1 January–29 February) Spring (1 March–31 May) Summer (1 June–31 August) Fall (1 September–30 September)	7 (5.8) 37 (30.8) 67 (55.8) 9 (7.5)	16 (6.7) 77 (32.1) 128 (53.3) 19 (7.9)	0.97
**Length of hospitalization (days) ± SD**	21.4 ± 21.4	17.2 ± 17.8	0.07

**Table 6:** Association of serum 25-hydroxy vitamin D with severe COVID-19 infection.

Predictor	Cases (*n* = 120) Serum 25(OH)D (ng/mL) ± SD	Controls (*n* = 240) Serum 25 (OH)D (ng/mL) ± SD	Beta Estimate	95% CI	*p*-Value
COVID-19 Hospitalization	23.9 ± 13.5	27.3 ± 15.4	−3.3	−6.3 to −0.4	0.03

## Data Availability

The datasets for this study are not publicly published due to the presence of potentially patient-identifiable information, but will be made available from the corresponding author on reasonable request.

## Abbreviations

25(OH)D	25-hydroxy-vitamin D
BMI	body mass index
CAMP	cathelicidin antimicrobial peptide
CI	confidence interval
OLS	ordinary least squares
SD	standard deviation
UCSD	University of California San Diego (UCSD)
